# Associations of Glycemic Index and Load With Coronary Heart Disease Events: A Systematic Review and Meta-Analysis of Prospective Cohorts

**DOI:** 10.1161/JAHA.112.000752

**Published:** 2012-10-25

**Authors:** Arash Mirrahimi, Russell J. de Souza, Laura Chiavaroli, John L. Sievenpiper, Joseph Beyene, Anthony J. Hanley, Livia S. A. Augustin, Cyril W. C. Kendall, David J. A. Jenkins

**Affiliations:** Clinical Nutrition and Risk Factor Modification Center, St. Michael's Hospital, Toronto, Ontario, Canada (A.M., R.J.D., L.C., J.L.S., L.S.A.A., C.W.C.K., D.J.A.J.); Division of Endocrinology, St. Michael's Hospital, Toronto, Ontario, Canada (D.J.A.J.); Department of Nutritional Sciences, Faculty of Medicine, University of Toronto, Toronto, Ontario, Canada (A.M., L.C., A.J.H., C.W.C.K., D.J.A.J.); Department of Medicine, Faculty of Medicine, University of Toronto, Toronto, Ontario, Canada (A.J.H., D.J.A.J.); Dalla Lana School of Public Health, Faculty of Medicine, University of Toronto, Toronto, Ontario, Canada (A.J.H., J.B.); Department of Nutrition, Harvard School of Public Health, Boston, MA (R.J.D.); Department of Pathology and Molecular Medicine, Faculty of Health Sciences, McMaster University, Hamilton, Ontario, Canada (J.L.S.); Department of Clinical Epidemiology and Biostatistics, Faculty of Health Sciences, McMaster University, Hamilton, Ontario, Canada (R.J.D., J.B.); Population Health Sciences Research Institute, Hospital for Sick Children, Toronto, Ontario, Canada (J.B.); College of Pharmacy and Nutrition, University of Saskatchewan, Saskatoon, Saskatchewan, Canada (C.W.C.K.)

**Keywords:** coronary heart disease, glycemic index and load, meta-analysis, nutrition, prospective cohort

## Abstract

**Background:**

Glycemic index (GI) and glycemic load (GL) have been associated with coronary heart disease (CHD) risk in some but not all cohort studies. We therefore assessed the association of GI and GL with CHD risk in prospective cohorts.

**Methods and Results:**

We searched MEDLINE, EMBASE, and CINAHL (through April 5, 2012) and identified all prospective cohorts assessing associations of GI and GL with incidence of CHD. Meta-analysis of observational studies in epidemiology (MOOSE) methodologies were used. Relative measures of risk, comparing the group with the highest exposure (mean GI of cohorts=84.4 GI units, range 79.9 to 91; mean GL of cohorts=224.8, range 166 to 270) to the reference group (mean GI=72.3 GI units, range 68.1 to 77; mean GL=135.4, range 83 to 176), were pooled using random-effects models, expressed as relative risk (RR) with heterogeneity assessed by χ^2^ and quantified by I^2^. Subgroups included sex and duration of follow-up. Ten studies (n=240 936) were eligible. Pooled analyses showed an increase in CHD risk for the highest GI quantile compared with the lowest, with RR=1.11 (95% confidence interval [CI] 0.99 to 1.24) and for GL, RR=1.27 (95% CI 1.09 to 1.49), both with evidence of heterogeneity (I^2^>42%, *P*<0.07). Subgroup analyses revealed only a significant modification by sex, with the female cohorts showing significance for GI RR=1.26 (95% CI 1.12 to 1.41) and for GL RR=1.55 (95% CI 1.18 to 2.03).

**Conclusions:**

High GI and GL diets were significantly associated with CHD events in women but not in men. Further studies are required to determine the relationship between GI and GL with CHD in men.

## Introduction

High-risk lifestyle and dietary patterns have been proposed to account for more than 80% of all coronary events in Western nations.^1^ The predominant concern in heart disease prevention has been saturated fatty acid (SFA) reduction, leading to widespread therapeutic adoption of low-total-fat, high-carbohydrate diets as the standard dietary approach for the reduction of coronary heart disease (CHD) risk.^2,3^ However, recent prospective cohort meta-analyses suggest an even greater increase in CHD risk when highly refined and readily absorbed carbohydrates replaced SFAs.^4,5^ As a result, SFAs per se no longer appeared to be associated with CHD, emphasizing the potentially deleterious effects of refined, rapidly absorbed carbohydrates.^4,5^ In addition, replacement of SFAs with unsaturated fatty acids and complex carbohydrates is associated with favorable changes in CHD risk factors.^6–8^ These findings have intensified the focus on carbohydrates, because diets rich in highly processed carbohydrates can lead to raised triglycerides (TGs),^9^ reductions in high-density lipoprotein cholesterol (HDL-C),^10^ and increasing CHD risk.^11^

Carbohydrates with differing physical form, particle size, chemical structure, and fiber content alter the rate of starch digestion and their physiological response. The glycemic index (GI) was developed to characterize the rate of digestion of a carbohydrate food compared with a reference carbohydrate food.^12^ Over the last 3 decades, clinical trials have demonstrated that reducing the GI or glycemic load (GL), the product of GI and the available carbohydrate content of a food,^13^ in the context of diets low in saturated fat, can improve CHD risk factors including body mass index (BMI), blood pressure, and serum cholesterol.^14–24^ Similar favorable effects have been seen with lower SFAs, higher poly-/monounsaturated fatty acids, and higher complex carbohydrate diets.^6–8^ These randomized controlled trials provide data that are harmonious with the emerging, albeit inconsistent, cohort literature on the unfavorable relationships between higher GI and GL dietary patterns and CHD risk. Cohort studies have also shown an association between low GI diets^25,26^ and reduced development of hyperglycemia and diabetes, further implicating the GI in the progression to CHD.^25,26^ This dietary pattern is also likely to have the additional advantage of reduced LDL cholesterol by lower intake of SFAs and dietary cholesterol.

Despite the proposed physiological mechanisms,^27^ published cohort studies in the last decade have produced mixed results for the associations of GI and GL with CHD.^28–31^ We therefore undertook a systematic review and meta-analysis of prospective cohort studies with healthy populations at baseline to determine whether associations exist between GI and GL with CHD.

## Methods

### Data Sources and Study Selection

We conducted separate searches for all prospective cohort studies that assessed potential associations between glycemic index or load and primary incidence of CHD (including myocardial infarction [MI] or death due to CHD) in adults. We followed the meta-analysis of observational studies in epidemiology (MOOSE) guidelines for this report.^32^ Electronic databases (MEDLINE 1946-March, Week 4, 2012; EMBASE 1980–2012, Week 13; CINAHL 1982–December 2011) were searched by 2 individuals independently; searches were supplemented by manual searches through the reference lists of original publications and review articles. The following search terms were used: ([Glycemic or Glycaemic Index] or [Glycemic or Glycaemic Load]) and (CHD or Cardiovascular Disease or CVD or MI) and (Prospective or Cohort). Titles and abstracts were initially reviewed to identify relevant reports by 2 independent reviewers (A.M., L.C., both investigators); reviewers conducted a subsequent full-text assessment of all studies in which there was uncertainty about the assessment of relevance. Disagreements regarding eligibility were resolved through discussion with 2 additional adjudicators (D.J.A.J/R.J.D.).

### Data Extraction

Two reviewers (A.M., L.C.) independently reviewed and extracted relevant data employing a standardized pro forma sheet with the first author and year of publication used as study identifiers for convenience. Data extracted from each cohort included information about sample size, population characteristics (age and sex), country of origin, follow-up duration, method of collecting dietary information, outcome measures, exposure quantification, and analytical methods, including adjustment parameters used for confounding factors. The most complete multivariate adjusted risk estimates from eligible studies assessing GI and GL associations with CHD events with their corresponding confidence intervals (CIs) were extracted to provide the main end points. All authors of eligible reports were contacted to acquire any missing data for each exposure level including number of events, person-years, mean or median dose of GI and GL (all values were converted to bread scale [GI=100] if not already reported as such,^31,33–36^ with bread scale=glucose scale/0.7),^37^ as well as risk estimates with corresponding CIs.

### Data Synthesis

Data were analyzed using Review Manager (RevMan) 5.1.4 (Cochrane Library software, Oxford, UK) and STATA version 11.0 (StataCorp, College Station, TX). The natural log-transformed relative risks of CHD events (including MI) with corresponding standard errors comparing the highest exposure level with the reference group from each cohort, irrespective of the number of quantile divisions in the original analysis, were pooled in separate analyses for GI and GL. The generic inverse variance method with random-effects models in RevMan were used to allow for heterogeneity assessment. Interstudy heterogeneity was tested by Cochrane's Q (χ^2^) and quantified by the I^2^ statistic. Regardless of *P* value, sensitivity analyses were performed to identify sources of heterogeneity.^38^ Potential publication bias was assessed visually by inspecting funnel plots of effect size against the standard error and formally tested using Begg's and Egger's tests in STATA.^39,40^ Our a priori stratified analyses included sex and duration of follow-up, that is, whether studies were more or less than 10 years, consistent with the 10-year Framingham Risk Score^41^ approach, and analyzed using meta-regression in STATA. Statistical significance was defined as *P*<0.05 for all comparisons, except for Cochrane's Q (χ^2^), where significance was set at <0.10.

## Results

### Search Results

**Figure 1.**
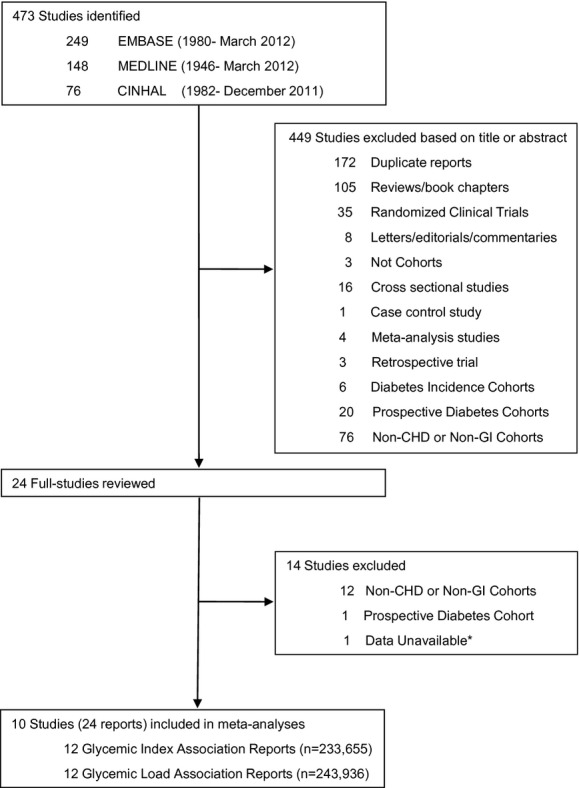
Literature search and review flow. CHD indicates coronary heart disease; GI, glycemic index.*The Hardy et al study, for which data were unavailable, only reported a rate of change in risk of CHD per 5 and 30 units of GL.

Figure 1 shows the flow of the literature applying the systematic search and selection strategies. In all, 473 eligible studies were identified by the search. A total of 10 studies with 12 GI reports^28–31,33,35,36,42,43^ and 12 GL reports^28–31,33–36,42,43^ were selected for analyses. Two reports^28,34^ on the Nurses' Health Study provided data on the GL exposure; only the report with the larger subject numbers and longer follow-up was included in the GL analyses.^34,44^

### Cohort Characteristics

The study characteristics and their diet compositions are shown in Tables 1 and 2, respectively. Of the 10 studies identified, 12 GI and 12 GL reports with CHD risk estimation were included in our analyses, with a total of 233 655^28–31,33,35,36,42,43^ and 240 936^28–31,33,34,35,36,42,43^ subjects, respectively. During 6 to 25 years of follow-up, 6940 coronary events were recorded. All studies used Cox proportional hazard models for CHD risk estimation analyses, except for 1 study^43^ which used restricted cubic spline models. The most common confounders adjusted for included age, BMI, and cigarette smoking, with full multivariate analyses outlined in Table 1. All cohorts excluded those with documented CHD or major CHD risk factor at time of enrollment, with the exception of 1 that included a population 5% of whom had diabetes^35^ but adjusted for diabetes status. All analyses were stratified by sex. The majority of the studies (7 of 10) used either a semiquantitative or quantitative Food Frequency Questionnaire (SFFQ or FFQ),^28,30,31,33,34,36,42^ but 3 used 4- or 7-day diet records or diet history interviews for food consumption patterns.^29,35,43^ All studies used the International Tables of Glycemic Index for assessing the GI of different foods; 3^28,29,34^ used the 1995 iteration,^45^ 6^30,31,33,36,42,43^ used the 2002 iteration,^46^ and 1^35^ used the 2008 iteration. One study^33^ further supplemented the 2002 International GI tables with GI values for 159 local food items that were tested at an academic institution following the International GI table methodologies. The majority of the included reports provided dietary pattern data for GL quantiles. The dietary patterns across the different quantiles of GL were similar between studies, with a trend of increasing carbohydrate and decreasing protein and fat content at higher GL quantiles (Table 2).

**Table 1.** Study Characteristics

Reference	N	Age Range	Country	Years Range	Mean Duration of Follow-Up, years	Number of Questionnaires Administered*	Total Number of New Events	Quantile Division	Mean/Median GI (GL) Interquantile Ranges	Method for Reporting Events	Adjustments†
Liu et al^28^	75 521 (W)	38 to 63	United States	1984–1994	10	3 (SFFQ)	761 CHD	Quintiles	68.1 to 82.7 (117 to 206)	Death certificates, medical/autopsy records	Hypertension; hypercholesterolemia; parental history of MI; menopause; use of multivitamins, vitamin E, and ASA; dietary intake of folate, vitamin E, trans fat, PUFA, and total protein

van Dam et al^29^	646 (M)	64 to 84	Netherlands	1985–1995	10	1 (Interview)	94 CHD	Tertiles	77.0 to 85.0 (161 to 230)	Death and hospital discharge registries	‡Prescribed diet (yesn/no); dietary intake (g/day) of PUFA and CH_2_O

Levitan et al^30^	36 246 (M)	45 to 79	Sweden	1998–2003	6	1 (FFQ)	1324 MI	Quartiles	73.0 to 82.9 (176 to 255)	Death and hospital discharge registries	Hypertension; hypercholesterolemia; parental history of MI; living alone; use of ASA; dietary intake of PUFA, total protein, CH_2_O

Beulens et al^31^	15 714 (W)	49 to 70	Netherlands	1993–2005	9	1 (FFQ)	556 CHD	Quartiles	68.6 to 81.4 (112 to 174)	Death and hospital discharge registries	Hypertension; SBP; menopause; education; use of vitamin E; dietary intake of PUFA, MUFA, total protein

Sieri et al^33^	13 637 (M)	35 to 64	Italy	1993–2004	7.9	1 (SFFQ)	305 CHD	Quartiles	71.9 to 80.7 (168.6 to 270)	Death certificates, hospital discharge registries, and clinical record	Hypertension; education

Sieri et al^33^	30 495 (W)	35 to 74	Italy	1993–2004	7.9	1 (SFFQ)	158 CHD	Quartiles	71.9 to 80.9 (175.4 to 265.4)	Death certificates, hospital discharge registries, and clinical record	Hypertension; education

Halton et al^34^	82 802 (W)	30 to 55	United States	1980–2000	20	6 (SFFQ)	1994 CHD	Deciles	GI N/A (83 to 256)	Death certificates, medical/autopsy records	Hypertension; hypercholesterolemia; parental history of MI; menopause; use of multivitamins, vitamin E, and ASA; dietary intake of trans fat, PUFA, MUFA, total protein

Mursu et al^35^	1981 (M)	42 to 60	Finland	1984–2005	16.1	1 (FR)	376 MI	Quartiles	70.4 to 89.0 (146.1 to 256.7)	Hospital discharge registries	Diabetes; SBP; hypertension medications; hypercholesterolemia; TAG; family history of CVD; education; dietary intake of folate, vitamin C, and PUFA

Burger et al^36^	8855 (M)	21 to 64	Netherlands	1993–2008	11.9	1 (FFQ)	581 CHD	Quartiles	72.9 to 85.0 (143.4 to 208.0)	Municipal administration registries, statistics Netherlands	Hypertension; education; dietary MUFA, PUFA, and energy-adjusted vitamin C, CH_2_O, and protein intake; plasma total cholesterol and HDL-C

Burger et al^36^	10 753 (W)	21 to 64	Netherlands	1993–2008	11.9	1 (FFQ)	300 CHD	Quartiles	73.3 to 84.9 (144.7 to 208.3)	Municipal administration registries, statistics Netherlands	Hypertension; education; dietary MUFA, PUFA, and energy-adjusted vitamin C, CH_2_O, and protein intake; plasma total cholesterol, and HDL-C

Levitan et al^42^	36 234 (W)	48 to 83	Sweden	1998–2006	9	1 (FFQ)	1138 MI	Quartiles	73.3 to 79.9 (128 to 188)	Death and hospital discharge registries	Hypertension; hypercholesterolemia; parental history of MI; menopause; education; marital status; use of multivitamin, vitamin E, and ASA; dietary intake of trans fat, PUFA, MUFA, total protein

Grau et al^43^	1684 (M)	30 to 70	Denmark	1974–1999	6 to 25	1 (Interview/FR)	NR	Quintiles	75.0 to 91.0 (102 to 220)	Hospital discharge registries	Education; energy-adjusted intake of fat, total protein, and CH_2_O

Grau et al^43^	1889 (W)	30 to 70	Denmark	1974–1999	6 to 25	1 (Interview/FR)	114 CHD	Quintiles	72.0 to 89.0 (84 to 166)	Hospital discharge registries	Education; energy-adjusted intake of fat, total protein, and CH_2_O

M indicates men; W, women; SFFQ, Semiquantitative Food Frequency Questionnaire (validated); FFQ, Food Frequency Questionnaire (validated); Interview, Diet History Interview; FR, food record; GI, glycemic index; GL, glycemic load; BMI, body mass index; CHD, coronary heart disease; MI, myocardial infarction; CVD, cardiovascular disease; HDL-C, high-density lipoprotein cholesterol; TAG, triacylglyceride; SBP, systolic blood pressure; ASA, aspirin; PUFA, polyunsaturated fatty acids; MUFA, monounsaturated fatty acids; CH_2_O, carbohydrate; NR, not reported; N/A, not available.

All studies used Cox proportional hazard models in their CHD risk estimation association analyses with GI and GL, except for Grau et al.^43^

All studies had a healthy starting population at the beginning of follow-up except for Mursu et al,^35^ who had a 5% diabetic population at the start.

* Number of Questionnaires Administered denotes number of administered questionnaires at the beginning of and throughout the study.

† All studies adjusted for age, BMI, physical activity, alcohol intake, total energy, saturated fat intake.

‡ Dietary fiber intake was adjusted for in all but 1 study.^29^

**Table 2.** Dietary Composition Patterns for Each GL Quantile by Total Energy (E), Percent Energy From Carbohydrates, Protein, and Fat and Types of Fat (SFA, PUFA, MUFA)*

Reference	First GL Quantile (CH_2_O:Prt:Fat)	Second GL Quantile (CH_2_O:Prt:Fat)	Third GL Quantile (CH_2_O:Prt:Fat)	Fourth GL Quantile (CH_2_O:Prt:Fat)	Fifth GL Quantile (CH_2_O:Prt:Fat)
Liu et al^28^ (W)	E: 1702 kcal/day	E: 1783 kcal/day	E: 1797 kcal/day	E: 1767 kcal/day	E: 1676 kcal/day
	
	(34:19:NR)	(38:17:NR)	(41:16:NR)	(45:15:NR)	(54:15:NR)
	
	SFA: 13	SFA: 12	SFA: 11	SFA: 11	SFA: 10
	
	PUFA: 7	PUFA: 6	PUFA: 6	PUFA: 6	PUFA: 5
	
	MUFA: 13	MUFA: 12	MUFA: 12	MUFA: 11	MUFA: 10
	
	Fiber/1000 kcal: 8.2 g	Fiber/1000 kcal: 9.0 g	Fiber/1000 kcal: 9.5 g	Fiber/1000 kcal: 9.6 g	Fiber/1000 kcal: 10.7 g

van Dam et al^29^ (M)	E: 2272 kcal/day	E: 2321 kcal/day	E: 2177 kcal/day	—	—
			
	(40:14:NR)	(42:14:NR)	(45:14:NR)		
			
	SFA: 17	SFA: 17	SFA: 18		
			
	PUFA: 6	PUFA: 6	PUFA: 6		
			
	MUFA: NR	MUFA: NR	MUFA: NR		
			
	Fiber/1000 kcal: 11.0 g	Fiber/1000 kcal: 10.9 g	Fiber/1000 kcal: 10.2 g		

Levitan et al^30^ (M)	E: 2703 kcal/day	E: 2728 kcal/day	E: 2710 kcal/day	E: 2705 kcal/day	—
		
	(35:14:NR)	(39:13:NR)	(42:13:NR)	(46:12:NR)	
		
	SFA: 13	SFA: 12	SFA: 11	SFA: 9	
		
	PUFA: 3	PUFA: 3	PUFA: 3	PUFA: 3	
		
	MUFA: 9	MUFA: 9	MUFA: 8	MUFA: 7	
		
	Fiber/1000 kcal: 5.0 g	Fiber/1000 kcal: 6.0 g	Fiber/1000 kcal: 6.53 g	Fiber/1000 kcal: 7.0 g	

Beulens et al^31^ (W)	E: 1797 kcal/day	E: 1828 kcal/day	E: 1819 kcal/day	E: 1789 kcal/day	—
		
	(36:16:37)	(41:16:35)	(45:15:34)	(51:15:31)	
		
	SFA: 16	SFA: 15	SFA: 14	SFA: 13	
		
	PUFA: 7	PUFA: 7	PUFA: 6	PUFA: 6	
		
	MUFA: 14	MUFA: 13	MUFA: 12	MUFA: 11	
		
	Fiber/1000 kcal: 11.1 g	Fiber/1000 kcal: 12.0g	Fiber/1000 kcal: 12.6 g	Fiber/1000 kcal: 13.4 g	

Sieri et al^33^ (M)	E: 2562 kcal/day	E: 2387 kcal/day	E: 2409 kcal/day	E: 2677 kcal/day	—
		
	(45:18:40)	(50:17:36)	(54:16:33)	(59:15:29)	
		
	SFA: 14	SFA: 12	SFA: 11	SFA: 10	
		
	PUFA: 5	PUFA: 4	PUFA: 4	PUFA: 4	
		
	MUFA: 19	MUFA: 17	MUFA: 16	MUFA: 14	
		
	Fiber/1000 kcal: 9.0 g	Fiber/1000 kcal: 10.1 g	Fiber/1000 kcal: 10.9 g	Fiber/1000 kcal: 13.1 g	

Sieri et al^33^ (W)	E: 2194 kcal/day	E: 1998 kcal/day	E: 2023 kcal/day	E: 2300 kcal/day	—
		
	(42:18:42)	(48:17:38)	(52:16:34)	(58:15:30)	
		
	SFA: 15	SFA: 13	SFA: 12	SFA: 10	
		
	PUFA: 5	PUFA: 4	PUFA: 4	PUFA: 4	
		
	MUFA: 20	MUFA: 18	MUFA: 16	MUFA: 14	
		
	Fiber/1000 kcal: 9.6 g	Fiber/1000 kcal: 10.5 g	Fiber/1000 kcal: 11.1 g	Fiber/1000 kcal: 12.0 g	

Halton et al^34^ (W)	NR	NR	NR	NR	NR

Mursu et al^35^ (M)	E:2490 kcal/day	E: 2310 kcal/day	E: 2306 kcal/day	E: 2494 kcal/day	—
		
	(36:16:42)	(42:16:40)	(45:15:37)	(49:15:35)	
		
	SFA: 20	SFA: 18	SFA: 17	SFA: 16	
		
	PUFA: 5	PUFA: 5	PUFA: 5	PUFA: 4	
		
	MUFA: 13	MUFA: 12	MUFA: 11	MUFA: 10	
		
	Fiber/1000 kcal: 8.8 g	Fiber/1000 kcal: 10.4 g	Fiber/1000 kcal: 11.3 g	Fiber/1000 kcal: 11.2 g	

Burger et al^36^ (W)	NA	NA	NA	NA	—

Burger et al^36^ (M)	NA	NA	NA	NA	—

Levitan et al^42^ (W)	E: 1765 kcal/day	E: 1727 kcal/day	E: 1728 kcal/day	E: 1745 kcal/day	—
		
	(41:18:NR)	(47:17:NR)	(50:16:NR)	(55:14:NR)	
		
	SFA: 17	SFA: 15	SFA: 13	SFA: 11	
		
	PUFA: 4	PUFA: 4	PUFA: 4	PUFA: 4	
		
	MUFA: NR	MUFA: NR	MUFA: NR	MUFA: NR	
		
	Fiber/1000 kcal: 10.8 g	Fiber/1000 kcal: 12.5 g	Fiber/1000 kcal: 13.4 g	Fiber/1000 kcal: 14.0 g	

Grau et al^43^ (M)	E: 2536 kcal/day	NR	E: 2608 kcal/day	NR	E: 2584 kcal/day
					
	(29:14:43)		(36:14:43)		(45:13:39)
					
	SFA: NR		SFA: NR		SFA: NR
					
	PUFA: NR		PUFA: NR		PUFA: NR
					
	MUFA: NR		MUFA: NR		MUFA: NR
					
	Fiber/1000 kcal: 5.9 g		Fiber/1000 kcal: 7.3 g		Fiber/1000 kcal: 8.1 g

Grau et al^43^ (W)	E: 1818 kcal/day	NR	E: 1867 kcal/day	NR	E: 1842 kcal/day
					
	(31:16:46)		(38:15:43)		(45:14:39)
					
	SFA: NR		SFA: NR		SFA: NR
					
	PUFA: NR		PUFA: NR		PUFA: NR
					
	MUFA: NR		MUFA: NR		MUFA: NR
					
	Fiber/1000 kcal: 6.6 g		Fiber/1000 kcal: 8.0 g		Fiber/1000 kcal: 8.7 g

GL indicates glycemic load; SFA, saturated fatty acids; PUFA, polyunsaturated fatty acids; MUFA, monounsaturated fatty acids; CH_2_O, carbohydrate; Prt, protein; NR, not reported; NA,^36^ author was contacted for data, but data were not available; E, total energy (kcal/day); Fiber, g/1000 kcal; W, women; M, men.

Percent energy for each component in each quantile was calculated from reported intake in grams multiplied by energy per gram (4 kcal/g for protein and CH_2_O, and 9 kcal/g for fat) and expressed as a percentage of the total energy in the respective quantile.

* Diet composition depicted in percent energy at every exposure level according to glycemic load quantiles.

### Glycemic Index and Coronary Heart Disease

**Figure 2.**
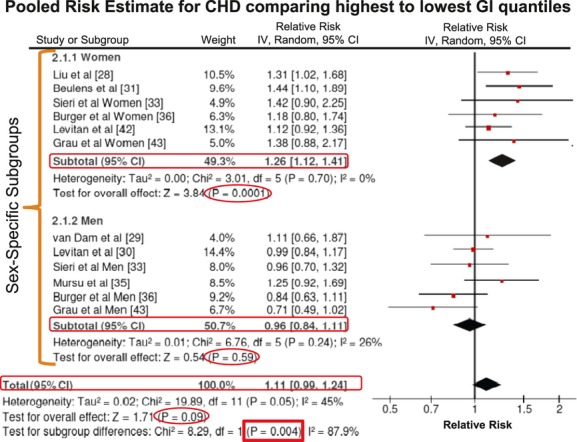
Pooled risk estimate of all prospective cohorts investigating the association of highest GI exposure with CHD events (including death and myocardial infarctions) relative to the reference exposure (ΔGI between mean of highest exposure and mean of reference=12.1±1.1 SE). The figure is stratified by sex-specific subgroups with subtotal boxes in 2.1.1 and 2.1.2 summarizing the pooled analysis for women^28,31,33,36,42,43^ and for men,^29,30,33,35,43^ respectively. The total analysis box represents the overall pooled analysis for both men and women. *P* values in circles are based on generic inverse variance (IV) methods in random-effects models and represent the significance for association of high-GI diets with CHD. The *P* value in a rectangle depicts the significance of differences between the subgroups. Interstudy heterogeneity was tested by Cochrane's Q (χ^2^) at a significance level of *P*<0.10 and quantified by I^2^.^38^ CHD indicates coronary heart disease; GI, glycemic index.

Figure 2 (overall analysis) shows the overall pooled relative risk estimation of GI with CHD events. The CHD incidence rate was increased at the highest level of GI exposure (mean GI of 84.4 GI units, range 79.9 to 91) relative to the lowest (mean GI of 72.3 GI units, range 68.1 to 77), RR=1.11, 95% CI 0.99 to 1.24, and approached significance (*P*=0.09) but with significant evidence of heterogeneity (I^2^=45%, *P*=0.05). Sensitivity analyses identified the Grau et al^43^ report on men as the largest contributor to heterogeneity. The removal of this study changed the risk estimate for the association of GI with CHD (RR=1.14 [95% CI 1.02 to 1.26], *P*=0.02) and also improved the precision of the estimate and eliminated much of the heterogeneity (I^2^=30%, *P*=0.16). A priori stratification revealed no significant modification of association for the duration of follow-up analysis with cohorts of ≥10 years^28,29,35,36,43^ (RR=1.08 [95% CI 0.89 to 1.31]) versus those of <10 years^30,31,33,42^ (RR=1.13 [95% CI 0.97 to 1.31]) as subsets (β=0.95 [95% CI 0.71 to 1.26]), both with evidence of heterogeneity (I^2^>44% for both, figure not shown). Sex, however, was a significant modifier of the association of GI with CHD (β=0.77 [95% CI 0.65 to 0.93]; *P*=0.004, Figure 2, sex-specific subgroups). The pooled female cohorts^28,31,33,36,42,43^ showed a larger, statistically significant association (ΔGI between mean of highest exposure and mean of reference=11.9±1.5 SE, RR=1.26 [95% CI 1.12 to 1.41]), whereas the male cohorts^29,30,33,35,36,43^ showed no association (ΔGI between mean of highest exposure and mean of reference=12.2±1.7 SE, RR=0.96 [95% CI 0.84 to 1.11]), with no significant evidence of heterogeneity in either subset.

### Glycemic Load and Coronary Heart Disease

**Figure 3.**
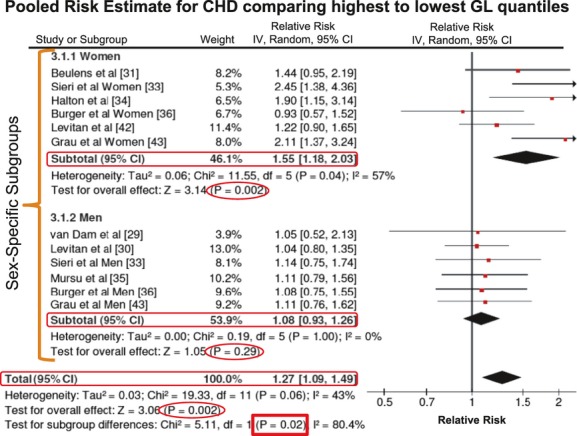
Pooled risk estimate of all prospective cohorts investigating the association of highest GL exposure with CHD events (including death and myocardial infarctions) relative to the reference exposure (ΔGL between mean of highest exposure and mean of reference=89.4±9.5 SE). The figure is stratified by sex-specific subgroups with subtotal boxes in 3.1.1 and 3.1.2 summarizing the pooled analysis for women^31,33,34,36,42,43^ and for men,^29,30,33,35,43^ respectively. The total analysis box represents the overall pooled analysis for both men and women. *P* values in circles are based on generic inverse variance (IV) methods in random-effects models and represent the significance for association of high-GL diets with CHD. The *P* value in a rectangle depicts the significance of differences between the subgroups. Interstudy heterogeneity was tested by Cochrane's Q (χ^2^) at a significance level of *P*<0.10 and quantified by I^2^.^38^ CHD indicates coronary heart disease; GI, glycemic index.

Figure 3 (overall analysis) shows the overall pooled relative risk estimation of GL with CHD events. The pooled risk estimation showed a significant increase in CHD risk (RR=1.27 [95% CI 1.09 to 1.49], *P*=0.002), with significant heterogeneity (I^2^=43%, *P*=0.06), for the highest level of GL exposure (mean GL of 224.8 GL units, range 166 to 270) relative to the lowest (mean GL of 135.4 GL units, range 83 to 176). Sensitivity analyses identified the Grau et al^43^ and Sieri et al^33^ reports on women as the largest contributors to heterogeneity when removed individually. The removal of Grau et al^43^ somewhat reduced the estimate of the association of GL with CHD (RR=1.21 [95% CI 1.05 to 1.38], *P*=0.007; I^2^=23%, *P*=0.23), as did the removal of Sieri et al^33^ (RR=1.22 [95% CI 1.06 to 1.40], *P*=0.005; I^2^=27%, *P*=0.19). Similar to the GI analyses, no modification in the association was revealed by the duration of follow-up analysis, ≥10 years^29,34–36,43^ (RR=1.26 [95% CI 1.01 to 1.59]) versus <10 years^30,31,33,42^ (RR=1.29 [95% CI 1.02 to 1.63]) with β=0.99 (95% CI 0.68 to 1.44), with evidence of heterogeneity (I^2^>47%, figure not shown) for both subgroups. Congruent with the GI analysis, sex was a significant modifier of the association of GL with CHD (β=0.73 [95% CI 0.56 to 0.96]; *P*=0.02, Figure 3, sex-specific subgroups). The female cohorts^31,33,34,36,42,43^ showed a larger, statistically significant association (ΔGL between mean of highest exposure and mean of reference=88.4±17.6 SE, RR=1.55 [95% CI 1.18 to 2.03]), whereas in the male cohorts,^29,30,33,35,36,43^ the association was not significant (ΔGL between mean of highest exposure and mean of reference=90.4±9.2 SE, RR=1.08 [95% CI 0.93 to 1.26]). There was significant evidence of heterogeneity in the female subset but not in the male subset.

### Publication Bias

**Figure 4.**
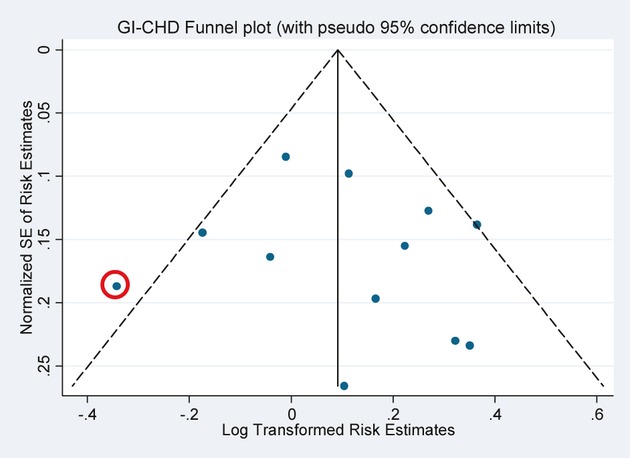
Test for publication bias in the overall pooled analysis of CHD risk estimates associated with the highest GI quantiles; Grau et al^43^ report on men was identified outside the 95% pseudo–confidence limits. Neither Begg's test (*P*>0.837) nor Egger's test (*P*=0.621) revealed evidence of publication bias.^39,40^ CHD indicates coronary heart disease; GI, glycemic index.

**Figure 5.**
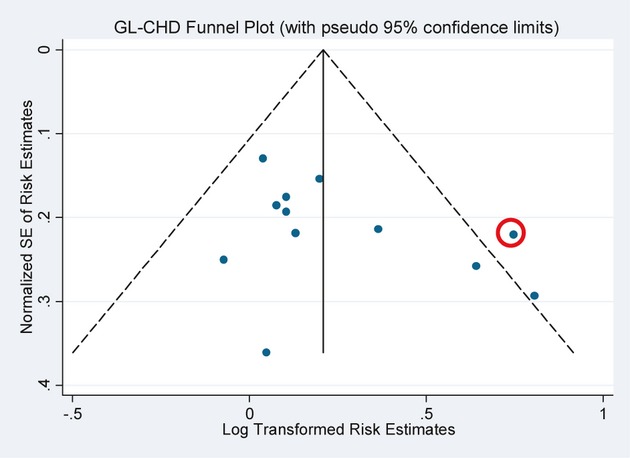
Test for publication bias in the overall pooled analysis of CHD risk estimates associated with highest GL quantiles; Grau et al^43^ report on women was identified outside the 95% pseudo–confidence limits. Begg's (*P*>0.115) and Egger's (*P*=0.134) tests approached significance for evidence of publication bias.^39,40^ CHD indicates coronary heart disease; GL, glycemic load.

Funnel plots for each of the overall analyses were inspected for presence of publication bias (Figures 4 and 5). Neither Begg's nor Egger's tests revealed significant evidence of publication bias in the overall analyses of GI and GL (*P*>0.115 for all). However, in the visual inspection of the GI funnel plot, the Grau et al^43^ report on men appears to be an outlier and in the GL funnel plot, and the Grau et al^43^ report on women appears to be an outlier (outside the pseudo 95% confidence limits).

## Discussion

We believe that this analysis represents the most comprehensive meta-analysis of the data presented as quantiles of GI and/or GL. However, we were unable to include 2 studies in this analysis because of the expression of results as either the substitution of low GI carbohydrates for SFAs, rather than dietary GI analysis, or the incremental association of dietary GI with CHD rather than quantile presentation (ie, CHD risk per 5 GI unit increments). These studies indicated that in Danish^47^ and Black American^44^ men, positive benefits for cardiovascular events were seen related to consumption of lower GI foods. We demonstrated an overall increased relative risk of CHD of 11% in the comparison of the highest versus lowest quantile of GI and a 27% increased relative risk of CHD for the highest versus the lowest quantile of GL. The effect was seen only in women with 26% increased relative risk of CHD for GI and 55% for GL.

The sex difference in the CHD response to the glycemic index was unexpected and may be the result of the larger total number of subjects in the female cohorts (n=177 887, CHD events=4260) than in the male cohorts (n=63 049, CHD events=2680) and our inability to include 2 studies, 1 from Denmark^47^ and 1 from the United States,^44^ both of which demonstrated adverse effects of high GI foods or diets on CHD outcomes in men. Furthermore, the 1 study^43^ of men in the present analysis that showed a near-significant deleterious effect of low-GI diets was also responsible for the heterogeneity in the analysis. This study differed from the other studies of men in several respects. The proportion (54%) of smokers was almost twice that of the other studies, and BMI was somewhat lower (25.3 versus 26.1 kg/m^2^); men with lower BMIs have been shown to be less susceptible to the effects of GI on CHD risk.^48^ There may also be another potential explanation for the difference between men and women, possibly a result of differences in diet reporting. For example, if women report more precisely, there would be less measurement error and hence better power. In addition, there may be more homogeneity (insufficient heterogeneity) in the diets of men and therefore a lack of the necessary power to detect potentially important relationships between dietary GI/GL and CHD risk in men.

Despite these reservations there are reasons why women may be potentially more vulnerable to high glycemic index diets. Part of the protection that women have from CHD may be related to their high HDL-C levels.^49,50^ Higher glycemic index diets tend to reduce circulating HDL-C concentrations and thus disproportionately increase CHD risk in women, especially when postmenopausal.^51^ At the same time, high-GI diets may raise TG levels,^52^ which may also carry more risk for CHD in women than in men.^53,54^

Other factors that in general may contribute to the increased CHD risk with high-GI diets are blood pressure and CRP, both of which may be raised by high-GI diets.^52,55^ Conversely, acarbose, the α-glucosidase inhibitor that converts dietary carbohydrate to a low glycemic index form, has been shown to prevent hypertension and CHD events in the STOP NIDDM trial.^56^

The link between low GI and GL diets and lower CHD risk is also substantiated by randomized clinical trials (RCTs) assessing the effects of dietary strategies low in GI and GL with low saturated fat content. A systematic review of such RCTs in overweight and obese subjects found that replacing refined carbohydrates with low-GI complex carbohydrates conferred more beneficial effects on CHD lipid risk factors when compared with complex starchy carbohydrates with higher GI.^57^ There has also been emerging evidence on the positive effects of low-GI diets on CHD risk factors such as oxidative damage^58^ and inflammation^22^ in overweight/obese and type 2 diabetic individuals, respectively. In addition, meta-analyses of low-GI RCTs have shown beneficial effects on body weight and lipid profiles in obese and overweight subjects^21^ as well as on glycemic control in type 2 diabetic subjects.^59^

The current studies do not suggest a latency effect of GI or GL on CHD risk. Because no studies have a time frame shorter than 6 years, it is not possible to determine whether the effect is early, possibly from alterations in clotting factors,^60^ or later due to reduction in the rate of atheroma formation secondary to oxidative damage.^61^

An earlier meta-analysis that assessed the effect of dietary GI on several health outcomes including diabetes, CHD, and cancer also concluded that low-GI diets were protective for diabetes, CHD, and colon and breast cancers.^62^ However, only 2 studies^28,29^ were available to assess CHD outcome at the time of that analysis.

In general, the glycemic index and glycemic load data were in agreement, although the magnitude of the CHD risk was greater based on the difference between the extreme quantiles of glycemic load. In the overall GL extreme quantile analysis there was significant heterogeneity that became nonsignificant when either the Grau et al^43^ or the Sieri et al^33^ reports on women were removed. The Grau et al^43^ report was visually an outlier on the publication bias funnel plot, which could potentially be a result of the difference in the analytical approach, using restricted cubic splines in Grau et al,^43^ compared with the methods of analyses used in other reports. We could not find an explanation for the heterogeneity in the overall GL analysis due to the Sieri et al^33^ report on women.

The weaknesses of the present study include the limited number of studies, the inability to include potentially relevant studies^44,47^ because of lack of necessary data, and the heterogeneity in the overall analyses, especially in the likely underpowered analyses of men. Another inherent limitation of observational analyses is the potential problem for residual confounding as well as the possibility of overadjusting, which remains an area of debate in epidemiology.^63^ Perhaps, the most common limitation in meta-analyses of dietary studies is the combination of dietary data collected using multiple instruments. Although no change was found in our overall conclusions with post hoc sensitivity analyses removing the 4^29,35,43^ reports with food diaries and interviews, because of the time frame of published reports, the GI and GL data were still compiled from various data sources. As such, our findings should be considered with caution, and further studies should be undertaken to allow sufficient power for subgroup analyses of dietary data sources.

Although the reports included men and women from Holland,^29,31,36^ Finland,^35^ Denmark,^43^ Sweden,^30,42^ Italy,^33^ and the United States,^28,34^ the majority of the population was white, limiting the racial diversity of this meta-analysis. However, it is noteworthy that our systematic review captured a report on African American men that, although not included because of lack of necessary data (ie, quantile analyses), showed that a 5-unit increase in GI conferred a 16% increase in CHD risk.^44^ Further studies are required with a wider range of ethnic groups and more racial diversity both in women and especially in men because cohorts of men were likely underpowered.

The strength of our study included the use of random-effects models to allow assessment of heterogeneity to guide the sensitivity analyses. We further believe that our systematic review was strengthened by our efforts to acquire as much data as possible by contacting all study authors to include all relevant studies in our analyses.

## Conclusion

We conclude that a reduction in the glycemic index and glycemic load may favorably affect CHD outcomes in women. Further studies are required to determine the effect of the glycemic index and load on CHD risk in men.

## References

[1] Stampfer MJ Hu FB Manson JE Rimm EB Willett WC Primary prevention of coronary heart disease in women through diet and lifestyle. N Engl J Med. 2000;343:16-22. 10882764 10.1056/NEJM200007063430103

[2] American Heart Association. Dietary guidelines for healthy American adults. A statement for physicians and health professionals by the Nutrition Committee, American Heart Association. Circulation. 1986;74:1465A-1468A. 3779925

[3] Krauss RM Deckelbaum RJ Ernst N Fisher E Howard BV Knopp RH Kotchen T Lichtenstein AH McGill HC Pearson TA Prewitt TE Stone NJ Horn LV Weinberg R Dietary guidelines for healthy American adults. A statement for health professionals from the Nutrition Committee, American Heart Association. Circulation. 1996;94:1795-1800. 8840887 10.1161/01.cir.94.7.1795

[4] Jakobsen MU O'Reilly EJ Heitmann BL Pereira MA Balter K Fraser GE Goldbourt U Hallmans G Knekt P Liu S Pietinen P Spiegelman D Stevens J Virtamo J Willett WC Ascherio A Major types of dietary fat and risk of coronary heart disease: a pooled analysis of 11 cohort studies. Am J Clin Nutr. 2009;89:1425-1432. 19211817 10.3945/ajcn.2008.27124 PMC2676998

[5] Siri-Tarino PW Sun Q Hu FB Krauss RM Meta-analysis of prospective cohort studies evaluating the association of saturated fat with cardiovascular disease. Am J Clin Nutr. 2010;91:535-546. 20071648 10.3945/ajcn.2009.27725 PMC2824152

[6] Mensink RP Zock PL Kester AD Katan MB Effects of dietary fatty acids and carbohydrates on the ratio of serum total to HDL cholesterol and on serum lipids and apolipoproteins: a meta-analysis of 60 controlled trials. Am J Clin Nutr. 2003;77:1146-1155. 12716665 10.1093/ajcn/77.5.1146

[7] Van Horn L McCoin M Kris-Etherton PM Burke F Carson JA Champagne CM Karmally W Sikand G The evidence for dietary prevention and treatment of cardiovascular disease. J Am Diet Assoc. 2008;108:287-331. 18237578 10.1016/j.jada.2007.10.050

[8] Appel LJ Sacks FM Carey VJ Obarzanek E Swain JF Miller ER III Conlin PR Erlinger TP Rosner BA Laranjo NM Charleston J McCarron P Bishop LM Effects of protein, monounsaturated fat, and carbohydrate intake on blood pressure and serum lipids: results of the omniheart randomized trial. JAMA. 2005;294:2455-2464. 16287956 10.1001/jama.294.19.2455

[9] Liu S Manson JE Stampfer MJ Holmes MD Hu FB Hankinson SE Willett WC Dietary glycemic load assessed by food-frequency questionnaire in relation to plasma high-density-lipoprotein cholesterol and fasting plasma triacylglycerols in postmenopausal women. Am J Clin Nutr. 2001;73:560-566. 11237932 10.1093/ajcn/73.3.560

[10] Mancini M Mattock M Rabaya E Chait A Lewis B Studies of the mechanisms of carbohydrate-induced lipaemia in normal man. Atherosclerosis. 1973;17:445-454. 4351720 10.1016/0021-9150(73)90034-8

[11] Jeppesen J Schaaf P Jones C Zhou MY Chen YD Reaven GM Effects of low-fat, high-carbohydrate diets on risk factors for ischemic heart disease in postmenopausal women. Am J Clin Nutr. 1997;65:1027-1033. 9094889 10.1093/ajcn/65.4.1027

[12] Jenkins DJ Wolever TM Taylor RH Barker H Fielden H Baldwin JM Bowling AC Newman HC Jenkins AL Goff DV Glycemic index of foods: a physiological basis for carbohydrate exchange. Am J Clin Nutr. 1981;34:362-366. 6259925 10.1093/ajcn/34.3.362

[13] Brand-Miller JC Thomas M Swan V Ahmad ZI Petocz P Colagiuri S Physiological validation of the concept of glycemic load in lean young adults. J Nutr. 2003;133:2728-2732. 12949357 10.1093/jn/133.9.2728

[14] Dickinson S Hancock DP Petocz P Ceriello A Brand-Miller J High-glycemic index carbohydrate increases nuclear factor-kappab activation in mononuclear cells of young, lean healthy subjects. Am J Clin Nutr. 2008;87:1188-1193. 18469238 10.1093/ajcn/87.5.1188

[15] Jenkins DJ Kendall CW McKeown-Eyssen G Josse RG Silverberg J Booth GL Vidgen E Josse AR Nguyen TH Corrigan S Banach MS Ares S Mitchell S Emam A Augustin LS Parker TL Leiter LA Effect of a low-glycemic index or a high-cereal fiber diet on type 2 diabetes: a randomized trial. JAMA. 2008;300:2742-2753. 19088352 10.1001/jama.2008.808

[16] Jenkins DJ Srichaikul K Kendall CW Sievenpiper JL Abdulnour S Mirrahimi A Meneses C Nishi S He X Lee S So YT Esfahani A Mitchell S Parker TL Vidgen E Josse RG Leiter LA The relation of low glycaemic index fruit consumption to glycaemic control and risk factors for coronary heart disease in type 2 diabetes. Diabetologia. 2011;54:271-279. 20978741 10.1007/s00125-010-1927-1 PMC3017317

[17] Luscombe ND Noakes M Clifton PM Diets high and low in glycemic index versus high monounsaturated fat diets: effects on glucose and lipid metabolism in NIDDM. Eur J Clin Nutr. 1999;53:473-478. 10403584 10.1038/sj.ejcn.1600779

[18] McMillan-Price J Petocz P Atkinson F O'Neill K Samman S Steinbeck K Caterson I Brand-Miller J Comparison of 4 diets of varying glycemic load on weight loss and cardiovascular risk reduction in overweight and obese young adults: a randomized controlled trial. Arch Intern Med. 2006;166:1466-1475. 16864756 10.1001/archinte.166.14.1466

[19] Pereira MA Swain J Goldfine AB Rifai N Ludwig DS Effects of a low-glycemic load diet on resting energy expenditure and heart disease risk factors during weight loss. JAMA. 2004;292:2482-2490. 15562127 10.1001/jama.292.20.2482

[20] Rizkalla SW Taghrid L Laromiguiere M Huet D Boillot J Rigoir A Elgrably F Slama G Improved plasma glucose control, whole-body glucose utilization, and lipid profile on a low-glycemic index diet in type 2 diabetic men: a randomized controlled trial. Diabetes Care. 2004;27:1866-1872. 15277409 10.2337/diacare.27.8.1866

[21] Thomas D Elliott E Baur L Low glycaemic index or low glycaemic load diets for overweight and obesity. Cochrane Database Syst Rev. 2007 CD005105 17636786 10.1002/14651858.CD005105.pub2 PMC9022192

[22] Wolever TM Gibbs AL Mehling C Chiasson JL Connelly PW Josse RG Leiter LA Maheux P Rabasa-Lhoret R Rodger NW Ryan EA The Canadian Trial of Carbohydrates in Diabetes (CCD), a 1-y controlled trial of low-glycemic-index dietary carbohydrate in type 2 diabetes: no effect on glycated hemoglobin but reduction in C-reactive protein. Am J Clin Nutr. 2008;87:114-125. 18175744 10.1093/ajcn/87.1.114

[23] Wolever TM Mehling C High-carbohydrate-low-glycaemic index dietary advice improves glucose disposition index in subjects with impaired glucose tolerance. Br J Nutr. 2002;87:477-487. 12010586 10.1079/BJNBJN2002568

[24] Larsen TM Dalskov SM van Baak M Jebb SA Papadaki A Pfeiffer AF Martinez JA Handjieva-Darlenska T Kunesova M Pihlsgard M Stender S Holst C Saris WH Astrup A Diet O Genes P Diets with high or low protein content and glycemic index for weight-loss maintenance. N Engl J Med. 2010;363:2102-2113. 21105792 10.1056/NEJMoa1007137 PMC3359496

[25] Salmeron J Manson JE Stampfer MJ Colditz GA Wing AL Willett WC Dietary fiber, glycemic load, and risk of non-insulin-dependent diabetes mellitus in women. JAMA. 1997;277:472-477. 9020271 10.1001/jama.1997.03540300040031

[26] Salmeron J Ascherio A Rimm EB Colditz GA Spiegelman D Jenkins DJ Stampfer MJ Wing AL Willett WC Dietary fiber, glycemic load, and risk of NIDDM in men. Diabetes Care. 1997;20:545-550. 9096978 10.2337/diacare.20.4.545

[27] Ludwig DS The glycemic index: physiological mechanisms relating to obesity, diabetes, and cardiovascular disease. JAMA. 2002;287:2414-2423. 11988062 10.1001/jama.287.18.2414

[28] Liu S Willett WC Stampfer MJ Hu FB Franz M Sampson L Hennekens CH Manson JE A prospective study of dietary glycemic load, carbohydrate intake, and risk of coronary heart disease in US women. Am J Clin Nutr. 2000;71:1455-1461. 10837285 10.1093/ajcn/71.6.1455

[29] van Dam RM Visscher AW Feskens EJ Verhoef P Kromhout D Dietary glycemic index in relation to metabolic risk factors and incidence of coronary heart disease: the Zutphen Elderly Study. Eur J Clin Nutr. 2000;54:726-731. 11002385 10.1038/sj.ejcn.1601086

[30] Levitan EB Mittleman MA Hakansson N Wolk A Dietary glycemic index, dietary glycemic load, and cardiovascular disease in middle-aged and older Swedish men. Am J Clin Nutr. 2007;85:1521-1526. 17556687 10.1093/ajcn/85.6.1521 PMC4355937

[31] Beulens JW de Bruijne LM Stolk RP Peeters PH Bots ML Grobbee DE van der Schouw YT High dietary glycemic load and glycemic index increase risk of cardiovascular disease among middle-aged women: a population-based follow-up study. J Am Coll Cardiol. 2007;50:14-21. 17601539 10.1016/j.jacc.2007.02.068

[32] Stroup DF Berlin JA Morton SC Olkin I Williamson GD Rennie D Moher D Becker BJ Sipe TA Thacker SB Meta-analysis of observational studies in epidemiology: a proposal for reporting. Meta-analysis of observational studies in epidemiology (MOOSE) group. JAMA. 2000;283:2008-2012. 10789670 10.1001/jama.283.15.2008

[33] Sieri S Krogh V Berrino F Evangelista A Agnoli C Brighenti F Pellegrini N Palli D Masala G Sacerdote C Veglia F Tumino R Frasca G Grioni S Pala V Mattiello A Chiodini P Panico S Dietary glycemic load and index and risk of coronary heart disease in a large Italian cohort: the EPICOR study. Arch Intern Med. 2010;170:640-647. 20386010 10.1001/archinternmed.2010.15

[34] Halton TL Willett WC Liu S Manson JE Albert CM Rexrode K Hu FB Low-carbohydrate-diet score and the risk of coronary heart disease in women. N Engl J Med. 2006;355:1991-2002. 17093250 10.1056/NEJMoa055317

[35] Mursu J Virtanen JK Rissanen TH Tuomainen TP Nykanen I Laukkanen JA Kortelainen R Voutilainen S Glycemic index, glycemic load, and the risk of acute myocardial infarction in Finnish men: the Kuopio Ischaemic Heart Disease Risk Factor Study. Nutr Metab Cardiovasc Dis. 2011;21:144-149. 19836217 10.1016/j.numecd.2009.08.001

[36] Burger KN Beulens JW Boer JM Spijkerman AM van der AD Dietary glycemic load and glycemic index and risk of coronary heart disease and stroke in Dutch men and women: the EPIC-MORGEN study. PLoS ONE. 2011;6:e25955 21998729 10.1371/journal.pone.0025955 PMC3187822

[37] Brouns F Bjorck I Frayn KN Gibbs AL Lang V Slama G Wolever TM Glycaemic index methodology. Nutr Res Rev. 2005;18:145-171. 19079901 10.1079/NRR2005100

[38] Higgins J Green S Cochrane handbook for systematic reviews of interventions version 5.0.0 [updated February 2008] 2008 The Cochrane Collaboration

[39] Begg CB Mazumdar M Operating characteristics of a rank correlation test for publication bias. Biometrics. 1994;50:1088-1101. 7786990

[40] Egger M Davey Smith G Schneider M Minder C Bias in meta-analysis detected by a simple, graphical test. BMJ. 1997;315:629-634. 9310563 10.1136/bmj.315.7109.629 PMC2127453

[41] Anderson KM Odell PM Wilson PW Kannel WB Cardiovascular disease risk profiles. Am Heart J. 1991;121:293-298. 1985385 10.1016/0002-8703(91)90861-b

[42] Levitan EB Mittleman MA Wolk A Dietary glycaemic index, dietary glycaemic load and incidence of myocardial infarction in women. Br J Nutr. 2010;103:1049-1055. 20003611 10.1017/S0007114509992674 PMC2851847

[43] Grau K Tetens I Bjornsbo KS Heitman BL Overall glycaemic index and glycaemic load of habitual diet and risk of heart disease. Public Health Nutr. 2011;14:109-118. 20576198 10.1017/S136898001000176X

[44] Hardy DS Hoelscher DM Aragaki C Stevens J Steffen LM Pankow JS Boerwinkle E Association of glycemic index and glycemic load with risk of incident coronary heart disease among whites and African Americans with and without type 2 diabetes: the Atherosclerosis Risk in Communities study. Ann Epidemiol. 2010;20:610-616. 20609341 10.1016/j.annepidem.2010.05.008 PMC3085981

[45] Foster-Powell K Miller JB International tables of glycemic index. Am J Clin Nutr. 1995;62:871S-890S. 7572722 10.1093/ajcn/62.4.871S

[46] Foster-Powell K Holt SH Brand-Miller JC International table of glycemic index and glycemic load values: 2002. Am J Clin Nutr. 2002;76:5-56. 12081815 10.1093/ajcn/76.1.5

[47] Jakobsen MU Dethlefsen C Joensen AM Stegger J Tjonneland A Schmidt EB Overvad K Intake of carbohydrates compared with intake of saturated fatty acids and risk of myocardial infarction: importance of the glycemic index. Am J Clin Nutr. 2010;91:1764-1768. 20375186 10.3945/ajcn.2009.29099

[48] Ding EL Malik VS Convergence of obesity and high glycemic diet on compounding diabetes and cardiovascular risks in modernizing China: an emerging public health dilemma. Global Health. 2008;4:4 18302739 10.1186/1744-8603-4-4 PMC2292178

[49] Knopp RH Paramsothy P Retzlaff BM Fish B Walden C Dowdy A Tsunehara C Aikawa K Cheung MC Gender differences in lipoprotein metabolism and dietary response: basis in hormonal differences and implications for cardiovascular disease. Curr Atheroscler Rep. 2005;7:472-479. 16256006 10.1007/s11883-005-0065-6

[50] Gordon DJ Probstfield JL Garrison RJ Neaton JD Castelli WP Knoke JD Jacobs DR Jr Bangdiwala S Tyroler HA High-density lipoprotein cholesterol and cardiovascular disease. Four prospective American studies. Circulation. 1989;79:8-15. 2642759 10.1161/01.cir.79.1.8

[51] Matthews KA Meilahn E Kuller LH Kelsey SF Caggiula AW Wing RR Menopause and risk factors for coronary heart disease. N Engl J Med. 1989;321:641-646. 2488072 10.1056/NEJM198909073211004

[52] Levitan EB Cook NR Stampfer MJ Ridker PM Rexrode KM Buring JE Manson JE Liu S Dietary glycemic index, dietary glycemic load, blood lipids, and C-reactive protein. Metabolism. 2008;57:437-443. 18249220 10.1016/j.metabol.2007.11.002 PMC2262400

[53] Reardon MF Nestel PJ Craig IH Harper RW Lipoprotein predictors of the severity of coronary artery disease in men and women. Circulation. 1985;71:881-888. 3986978 10.1161/01.cir.71.5.881

[54] Austin MA Hokanson JE Edwards KL Hypertriglyceridemia as a cardiovascular risk factor. Am J Cardiol. 1998;81:7B-12B. 9526807 10.1016/s0002-9149(98)00031-9

[55] Liu S Manson JE Buring JE Stampfer MJ Willett WC Ridker PM Relation between a diet with a high glycemic load and plasma concentrations of high-sensitivity C-reactive protein in middle-aged women. Am J Clin Nutr. 2002;75:492-498. 11864854 10.1093/ajcn/75.3.492

[56] Chiasson JL Josse RG Gomis R Hanefeld M Karasik A Laakso M STOP-NIDDM Trial Research Group. Acarbose treatment and the risk of cardiovascular disease and hypertension in patients with impaired glucose tolerance: the STOP-NIDDM trial. JAMA. 2003;290:486-494. 12876091 10.1001/jama.290.4.486

[57] Gastrich M Lasser N Wien M Bachmann G Dietary complex carbohydrates and low glycemic index/load decrease levels of specific metabolic syndrome/cardiovascular disease risk factors. Top Clin Nutr. 2008;23:76-96.

[58] Botero D Ebbeling CB Blumberg JB Ribaya-Mercado JD Creager MA Swain JF Feldman HA Ludwig DS Acute effects of dietary glycemic index on antioxidant capacity in a nutrient-controlled feeding study. Obesity (Silver Spring). 2009;17:1664-1670. 19543205 10.1038/oby.2009.203 PMC2752149

[59] Thomas D Elliott EJ Low glycaemic index, or low glycaemic load, diets for diabetes mellitus. Cochrane Database Syst Rev. 2009 CD006296 19160276 10.1002/14651858.CD006296.pub2 PMC6486008

[60] Jarvi AE Karlstrom BE Granfeldt YE Bjorck IE Asp NG Vessby BO Improved glycemic control and lipid profile and normalized fibrinolytic activity on a low-glycemic index diet in type 2 diabetic patients. Diabetes Care. 1999;22:10-18. 10333897 10.2337/diacare.22.1.10

[61] Hu Y Block G Norkus EP Morrow JD Dietrich M Hudes M Relations of glycemic index and glycemic load with plasma oxidative stress markers. Am J Clin Nutr. 2006;84:70-76 quiz 266-267 16825683 10.1093/ajcn/84.1.70

[62] Barclay AW Petocz P McMillan-Price J Flood VM Prvan T Mitchell P Brand-Miller JC Glycemic index, glycemic load, and chronic disease risk – a meta-analysis of observational studies. Am J Clin Nutr. 2008;87:627-637. 18326601 10.1093/ajcn/87.3.627

[63] Schisterman EF Cole SR Platt RW Overadjustment bias and unnecessary adjustment in epidemiologic studies. Epidemiology. 2009;20:488-495. 19525685 10.1097/EDE.0b013e3181a819a1 PMC2744485

